# Adiponectin is protective against endoplasmic reticulum stress-induced apoptosis of endothelial cells in sepsis

**DOI:** 10.1590/1414-431X20187747

**Published:** 2018-11-14

**Authors:** Yun Hou, Xi Feng Wang, Zhi Qiang Lang, Yin Chuan Jin, Jia Rong Fu, Xiao Min Xv, Shi Tian Sun, Xin Xin, Lian Shuang Zhang

**Affiliations:** 1 Department of Histology and Embryology, Binzhou Medical University, Yan Tai, China; 2 Department of Critical Care Medicine, Yu Huang Ding Hospital, Qingdao University, Yan Tai, China; 3 Department of Pathology, Yu Huang Ding Hospital, Qingdao University, Yan Tai, China; 4 College of Clinical Medicine, Bin Zhou Medical University, Yan Tai, China

**Keywords:** Adiponectin, Apoptosis, Endoplasmic reticulum stress, Endothelial cell, Sepsis

## Abstract

Endoplasmic reticulum (ER) stress is a critical molecular mechanism involved in the pathogenesis of sepsis. Hence, strategies for alleviating this stress may be essential for preventing cardiovascular injuries under sepsis. Adiponectin is secreted by adipocytes and its levels are decreased in sepsis. The purpose of this study was to investigate the protective effects of adiponectin treatment on endothelial cells and its mechanism. Male Wistar rats underwent cecal ligation and puncture (CLP) before being treated with adiponectin (72 and 120 μg/kg). The levels of malondialdehyde (MDA) in plasma, histological structure, and apoptosis of endothelial cells were evaluated. *In vitro*, human umbilical vein endothelial cells (HUVECs) were treated with adiponectin at 10 and 20 μg/mL for 24 h after stimulation by lipopolysaccharide (LPS). The levels of reactive oxygen species (ROS), ultrastructure, rate of apoptosis, the expression of inositol-requiring enzyme 1α (IRE1α) protein, and its downstream molecules (78 kDa glucose-regulated protein (GRP78), C/EBP homologous protein (CHOP), and caspase-12) were detected. The results showed that the levels of MDA and ROS induced by CLP or LPS stimulation were increased. Furthermore, endothelial cell apoptosis was increased under sepsis. The IRE1α pathway was initiated, as evidenced by activated IRE1α, increased GRP78, and up-regulated CHOP and caspase-12 in HUVECs. Following treatment with adiponectin, the number of apoptotic endothelial cells was markedly decreased. These findings demonstrated that treatment with adiponectin decreased apoptosis of endothelial cells caused by sepsis by attenuating the ER stress IRE1α pathway activated by oxidative stress.

## Introduction

Sepsis is a life-threatening clinical condition characterized by organ dysfunction and shock (1). Organ dysfunction is closely related to blood circulation disorders. Septic patients commonly suffer from a dysregulated and activated coagulation system. The occurrence of coagulation dysfunction is related to endothelial cells that are located in the innermost layer of blood vessels, making them one of the primary targets of the inflammatory response during sepsis (2). Lipopolysaccharide (LPS) and other inflammatory factors in blood stimulate endothelial cells (3) and result in endothelial cell injury (4). Previous studies have shown that some proinflammatory cytokines, especially tumor necrosis factor-α, interleukin (IL)-1, and procoagulants such as tissue factor, intercellular adhesion molecule-1, and vascular cell adhesion molecule-1 are released from damaged endothelium (5,6) and further promote coagulation disorders. Together, these can induce disseminated intravascular coagulation and eventually lead to multiple organ failure (7,8).

Endoplasmic reticulum (ER) is an important organelle for cellular protein synthesis and processing, maintaining stability of the intracellular environment. ER stress plays an important role in the pathological states of sepsis (9,10). Thus, it is crucial to investigate novel interventions to improve the endothelial injury induced by ER stress, and prevent coagulation disorders in severely septic patients. Sepsis also accompanies oxidative stress, which induces the formation of reactive oxygen species (ROS) and malondialdehyde (MDA), a marker of lipid peroxidation (11). The acceleration of ER malfunction is associated with oxidative stress (12,13).

Adiponectin is a serum adipokine secreted primarily by adipocytes. It is associated with obesity, atherosclerosis, and coronary artery disease (14). Apart from its antioxidant and anti-inflammatory activities in some disease states (15), previous studies have shown that adiponectin can improve the function of vascular endothelium (16). Moreover, recent research showed that adiponectin promoted the proliferation of bovine mammary epithelial cells via inhibiting ER stress responses (17).

Adiponectin has different oligomeric isoforms, including trimeric, hexameric, and the high molecular weight (HMW) oligomeric complex (18). Septic patients exhibit low levels of total adiponectin (19) and a significant increase in total and HMW adiponectin, which is related to clinical recovery from sepsis (20) (Supplementary Table S1). However, few reports focus on whether adiponectin can protect the endothelium and is involved in the mechanisms of sepsis. Hence, the purpose of this study was to investigate the protective effects of adiponectin treatment on alleviating apoptosis of endothelial cells and reveal its related mechanism about oxidative stress and ER stress in rat and human umbilical vein endothelial cell (HUVECs) models of sepsis.

## Material and Methods

### Design of *in vivo* experiments

All the animal experiments were carried out in accordance with the Guide for the Care and Use of Laboratory Animals by the National Institutes of Health and the Ethical Committee of Binzhou Medical University. Eight-week-old Wistar rats weighing 250 to 300 g were maintained on a 12-h light/dark cycle at 22±3°C. The animals were allowed free access to food and water. After one week of acclimation, the animals were randomly divided into 4 groups of 12 rats each: sham, cecal ligation and puncture (CLP), CLP + adiponectin (72 μg/kg) treatment, and CLP + adiponectin (120 μg/kg) treatment. The CLP model was generated using the method described in our previous experiments (21). The dose of adiponectin was selected based on the results of pre-experiments.

The rats in the sham group received 120 μg/kg of saline intravenously (*iv*), 12 h after undergoing a sham operation consisting of laparotomy and bowel manipulation but no CLP; the rats in the CLP group received 120 μg/kg saline *iv*, 12 h after undergoing CLP; and the rats in the adiponectin treatment groups 72 or 120 μg/kg of adiponectin *iv* after undergoing CLP. After 24 h, the rats were sacrificed and the abdominal aorta was removed and fixed until subsequent analysis.

### Measurement of plasma MDA

The concentration of MDA was determined using an MDA kit (Jiancheng Biotechnology Company, China). All the reagents and samples were prepared according to the manufacturer's protocol. Absorbance was measured at 532 nm with a microplate reader (DNM-9602G, Perlong Medical Equipment, China).

### TdT-mediated dUTP nick end labeling (TUNEL)

The apoptosis of endothelial cells in the tissue sections was detected by the terminal deoxyribonucleotide transferase-mediated nick-end labeling (TUNEL) assay kit (Keygen, China), according to the manufacturer's instructions. The slides were placed in DAB for 5 min and stained with hematoxylin. These analyses were performed under a light microscope at 400× magnification with 15 different fields using computer-aided software (Olympus X71-F22PH, Japan). The gray values of the apoptotic cells were quantified by computer-assisted image analysis (Leica LAS Image Analysis V4.0, Germany).

### Cell culture and design of *in vitro* experiments

Human umbilical vein endothelial cells (HUVECs) were purchased from ATCC Manassas (No. CRL-1730, USA). The cells were cultured in a complete medium consisting of DMEM (HyClone, USA), supplemented with 10% fetal bovine serum (Gibco, USA), 100 U/mL of penicillin, and 100 μg/mL of streptomycin.

The cells were divided into control, LPS stimulation, and LPS stimulation + adiponectin treatment groups. LPS (1 μg/mL of LPS for 12 h) was added to the culture medium of the LPS stimulation group; the LPS stimulation + adiponectin treatment groups were treated with 10 μg/mL or 20 μg/mL of adiponectin for 24 h after exposure to 1 μg/mL of LPS for 12 h. To explore the role of oxidative stress and the inositol-requiring enzyme 1α (IRE1α) pathways at the cellular level, the HUVECs were pretreated with a ROS inhibitor (NAC, 1 mM) and an IRE1α inhibitor (STF-083010, 10 μM) for 4 h prior to LPS exposure. An equivalent volume of DMEM was added to the control group. Five replicate wells were used for each treatment group.

### Laser confocal microscopy

The cells were incubated with 2,7-dichlorofluorescein-diacetate (DCFH-DA, 50 ng/mL, Jiancheng, China) at 37°C for 30 min in the dark. The cells were subsequently washed with PBS and observed under a confocal laser scanning microscope (Leica), and the images were captured. The mean fluorescence intensity of dichlorofluorescein (DCF) was analyzed.

### Western blotting

The cell protein lysates were electrophoretically separated and transferred to polyvinylidene fluoride (PVDF) membranes. After blocking, the PVDF membranes were incubated overnight at 4°C with IRE1α (1:800), p-IRE1α (1:600), caspase 12 (1:800), C/EBP homologous protein (CHOP, 1:1000), and 78 kDa glucose-regulated protein (GRP78, 1:1000), all rabbit antibodies (CST, USA). The membranes were then incubated with anti-rabbit IgG secondary antibody (CST). After washing, the proteins bound to the antibody were measured by a chemiluminescent reagent (Thermo Fisher Scientific, USA). Densitometric analyses were performed using the Image Lab™ Software (Bio-Rad Laboratories Inc., USA).

### Laser confocal microscopy and flow cytometry

The LPS-induced apoptosis of cells was observed by an apoptosis detection kit (Keygen), according to the manufacturer's protocols. The cells were stained with FITC-conjugated annexin V and propidium iodide (PI) and observed under a confocal laser scanning microscope (Leica).

To evaluate the rate of apoptosis, the HUVECs were stained with both FITC-conjugated annexin V and PI, and analyzed by an apoptosis detection kit (KeyGen). The cells incubated with FITC-conjugated annexin V and PI were analyzed by a flow cytometer (Millipore, USA). The different populations of cells were identified by the different labeling patterns in the Annexin V-PI analysis.

### Transmission electron microscopy

Transmission electron microscopy (TEM) was performed according to experimental procedures. The cells were fixed by immersing in Karnovsky's fixative at 4°C after washing and dehydrating in increasing concentrations of ethanol, after which the cells were embedded in Epon using Beem capsules (Germany). Ultrathin 60-nm thick sections were cut and stained with lead citrate and uranyl acetate and viewed under a transmission electron microscope (JEM 2100, Japan).

### Statistical analysis

Data were analyzed using the SPSS software, version 17.0 (USA). The *in vivo* differences among groups were analyzed by the Kruskal-Wallis test, while the *in vitro* differences were analyzed by a one-way analysis of variance (ANOVA) followed by the Fisher's least significant difference (LSD) test. The data are reported as means±SE. The results were considered significant at P<0.05.

## Results

### Treatment with adiponectin downregulated plasma levels of MDA

**Figure 1.**
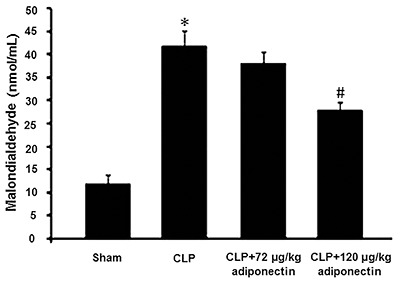
Plasma malondialdehyde (MDA) measured in Wistar rats by MDA kit at 24 h after cecal ligation and puncture (CLP) (n=8) and Sham (n=8) surgeries. Data are reported as means±SE. *P<0.05 compared to the sham group, ^#^P<0.05 compared to the CLP group (Kruskal-Wallis test).

The plasma levels of MDA were significantly higher in the CLP group than those of the sham group; however, there was no difference in the plasma levels of MDA between the adiponectin (72 μg/kg) treatment group and the CLP group (P>0.05). Compared to the plasma levels of MDA in the CLP group, those of the adiponectin (120 μg/kg) treatment group were significantly lower (P<0.05) (Figure 1).

### Adiponectin treatment alleviated the apoptosis of endothelial cells in vascular tissue

**Figure 2.**
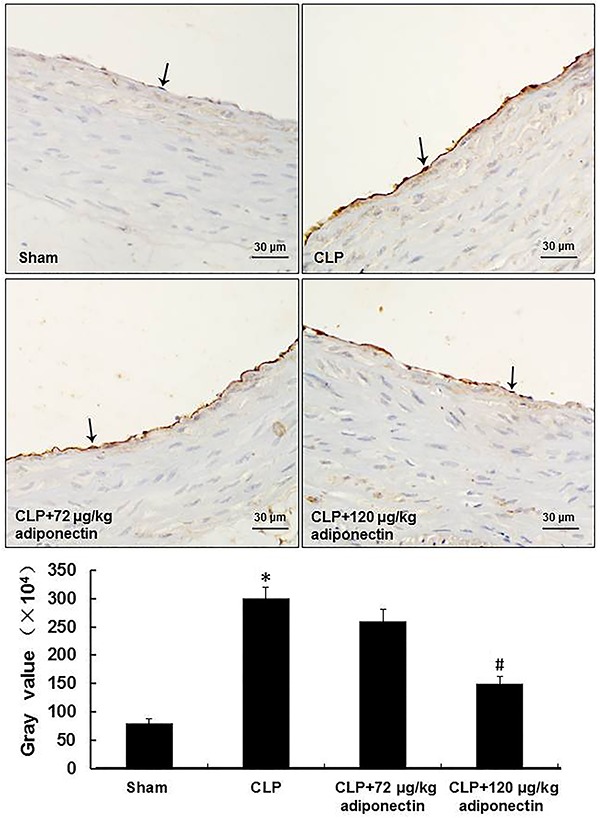
Effects of adiponectin on the expressions of TUNEL positive cells in each group. *Top panel*, The arrows indicate TUNEL positive cells. *Bottom panel*, Gray values of the TUNEL positive cells in the four groups. Scale bar: 30 μm. *P<0.05 compared to the sham group, ^#^P<0.05 compared to the cecal ligation and puncture (CLP) group (Kruskal-Wallis test).

The results showed that the immunopositive TUNEL staining was observed in the endothelial cells of all groups (Figure 2). The number of TUNEL-positive cells was significantly higher in the CLP group compared to that of the sham group (P<0.05). However, the number of TUNEL-positive cells decreased significantly in the adiponectin (120 μg/kg) treatment group compared to the CLP group (Figure 2).

### Adiponectin treatment downregulated the levels of ROS in HUVECs

**Figure 3.**
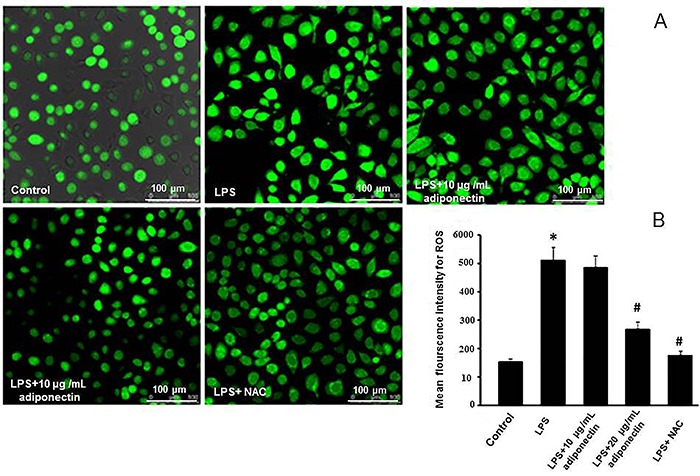
Effects of adiponectin on reactive oxygen species (ROS) levels in human umbilical vein endothelial cells (HUVECs). Data are reported as means±SE (n=5). *A*, Green fluorescence shows the expressions of ROS. Scale bar: 100 μm. *B*, Average fluorescence intensity of ROS in each group. *P<0.05 compared to the control group, ^#^P<0.05 compared to the LPS group (ANOVA).

The intracellular levels of ROS were determined using the DCFH-DA probe (Figure 3A). As shown in Figure 3B, ROS significantly increased in the LPS group compared to the control group. The reduction in the expression of ROS was significant in the LPS + 20 μg/mL adiponectin group compared to the LPS group (P<0.05). Additionally, NAC decreased the production of ROS in the LPS-stimulated HUVECs (P<0.05).

### Adiponectin treatment altered the ultrastructure of HUVECs

**Figure 4.**
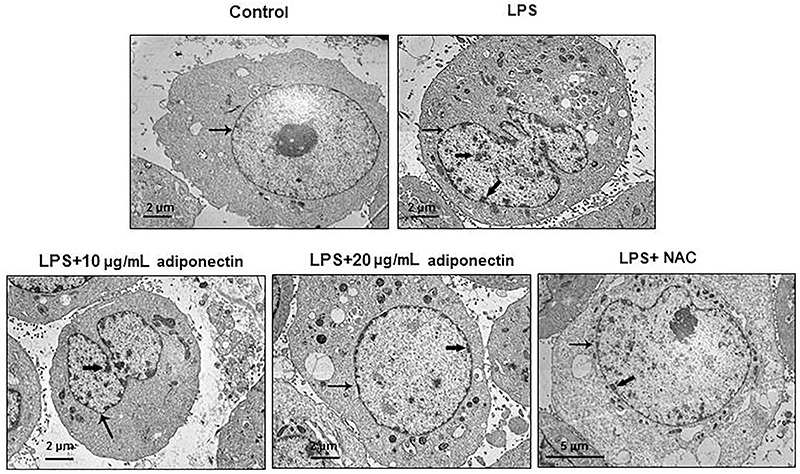
Electron micrographs of human umbilical vein endothelial cells (HUVECs) in each group. To explore the role of the inositol-requiring enzyme 1α (IRE1α) pathway at the cellular level, HUVECS were pretreated with IRE1α inhibitor (STF-083010, 10 μM) for 4 h before lipopolysaccharide (LPS) exposure. Thin black arrows indicate nuclear membrane; thick black arrows indicate chromatin aggregation. Scale bar: 2 μm.

To gain more information on an ultrastructural level, TEM was used to observe the ultrastructure of the HUVECs. As shown in Figure 4, the cell membrane and nuclei appeared clear in the control group. However, in the LPS group and adiponectin (10 μg/mL) treatment group, the chromatin appeared fragmented, as indicated by the thick arrow (Figure 4). Chromatin aggregation improved following treatment with adiponectin, and was only aggregated in the inner side of the nuclear membrane in the adiponectin (20 μg/mL) treatment group and the STF-083010 (IRE1α inhibitor)-treated group.

### Treatment with adiponectin reduced apoptosis by inhibiting the activation of IRE1**α** in the HUVECs

**Figure 5.**
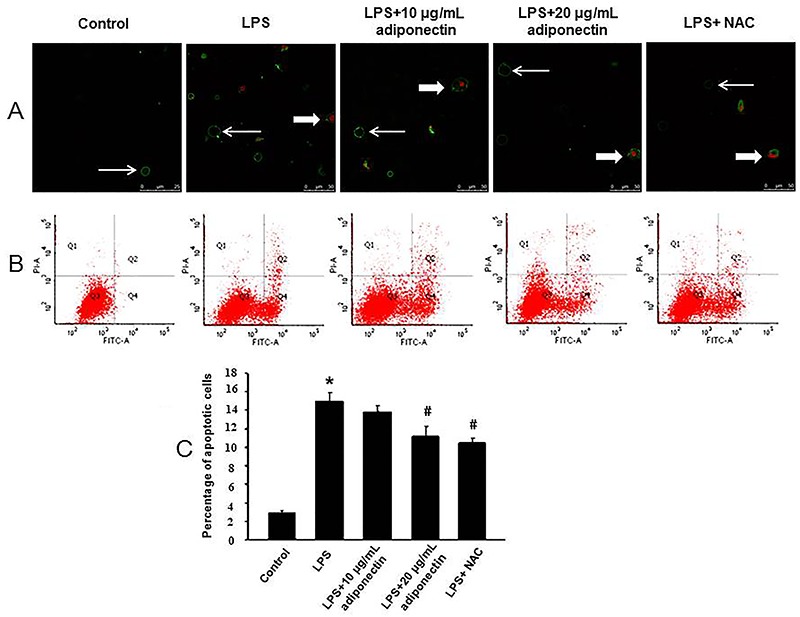
Effects of adiponectin on the apoptosis of human umbilical vein endothelial cells (HUVECs). *A*, Apoptotic cells under the confocal laser scanning microscope. Thin arrows indicate early apoptotic cells, thick arrows indicate late apoptotic cells. *B*, The percentage of both early and late apoptotic cells was measured by flow cytometry. *C*, Percentage of apoptotic cells was measured. To explore the role of IRE1α pathways at the cellular level, HUVECS were pretreated with IRE1α inhibitor (STF-083010, 10 μM) for 4 h before LPS exposure. Five replicate wells were used in each treatment group. *P<0.05 compared to the control group, ^#^P<0.05 compared to the lipopolysaccharide (LPS) group (ANOVA).

The early apoptotic cells were stained with green fluorescent dye (indicated by thin arrows) and the late apoptotic cells were stained with green and red fluorescent dyes (indicated by thick arrows) (Figure 5A). The results of flow cytometry indicated that there was a significant increase in the percentage of apoptotic cells in the LPS group compared to the control group, and a significant decrease in the adiponectin (20 μg/mL) treatment group (P<0.05) compared to the LPS group. Moreover, treatment with the IRE1α inhibitor (STF-083010, 10 μM) significantly decreased the percentage of LPS-induced apoptotic cells (P<0.05) (Figure 5B and C).

### Treatment with adiponectin altered the expression of proteins involved in the IRE1**α** pathway in the HUVECs

**Figure 6.**
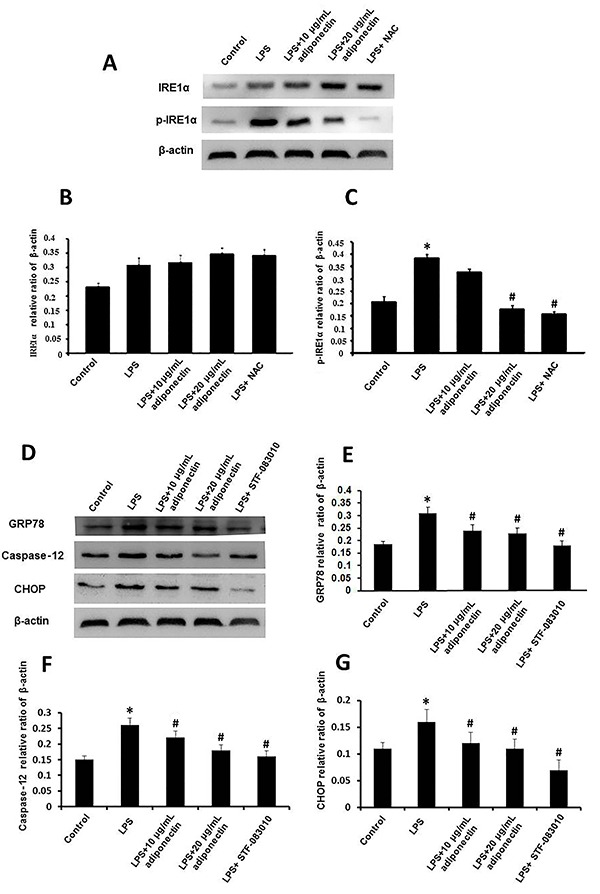
Effects of adiponectin on the protein expression of human umbilical vein endothelial cells (HUVECs). Expressions of inositol-requiring enzyme 1α (IRE1α), p-IRE1α, 78 kDa glucose-regulated protein (GRP78), caspase-12, and C/EBP homologous protein (CHOP) (*A and D*) in each group. Relative expressions of IRE1α, p-IRE1α, GRP78, caspase12, and CHOP (*B*, *C*, *E*, *F*, *G*) in each group were measured. To explore the role of IRE1α pathways at the cellular level, HUVECS were pretreated with IRE1α inhibitor (STF-083010, 10 μM) for 4 h before LPS exposure. An equivalent volume of DMEM was added to the control group. Five replicate wells were used in each treatment group. Data are reported as means±SE. *P<0.05 compared to the control group, ^#^P<0.05 compared to the LPS group (ANOVA).

The expression of IRE1α remained unaltered in all groups (P>0.05). The expression of p-IRE1α significantly increased in the LPS group compared to the control group (P<0.05), but decreased significantly in the adiponectin (20 μg/mL) treatment group (P<0.05), compared to the LPS group. Moreover, treatment with NAC, the inhibitor of oxidative stress, significantly decreased the expression of p-IRE1α in the LPS-treated HUVECs (P<0.05, Figure 6A, B, and C). The expression of GRP78, CHOP, and caspase-12 increased significantly in the LPS group compared to the control group (P<0.05). However, treatment with adiponectin and STF-083010 significantly decreased the expressions of GRP78, CHOP, and caspase-12 compared to those after LPS treatment (P<0.05, Figure 6D, E, F, and G).

## Discussion

Coagulation dysfunction under sepsis has become the major factor affecting the prognosis of critically ill patients in intensive care units. Endothelial cells are related to coagulation because they produce numerous factors, such as tissue factor, that affect this process (8,22). Coagulation cascades triggered by endothelial damage can induce microcirculatory dysfunction that is the major mechanism in sepsis (8). As such, it is important to understand the endothelial cell injury occurring under sepsis and investigate potential therapies.

Adiponectin is secreted from adipose tissue, and endogenous adiponectin exerts protective effects against hypertensive vascular injury (23). Previous studies reported that the adiponectin concentration was lower in some cardiovascular diseases (24), and was reduced in mice subjected to CLP (25). One recent study showed that mortality was increased in adiponectin-null mice following CLP (26), (Table S1), and clinical recovery from sepsis, which is related to an increase in total and HMW adiponectin, was investigated (20). Thus, together, these studies indicate that exogenous supplementation with adiponectin may have protective effects on sepsis.

In our study, sepsis was induced in rats by CLP, which closely resembles the clinical pathophysiologic course of sepsis (27). Our results showed that endothelial cell injury and apoptosis were induced following CLP. This injury was characterized by nuclear pyknosis and exfoliation from the vessel wall. In addition, the number of cells stained positive with the TUNEL assay was increased. Exogenous adiponectin at a dose of 120 μg/kg attenuated endothelial cell apoptosis in the septic rats.

*In vitro*, HUVECs apoptosis was induced by LPS stimulation. The specific manifestations were the appearance of chromatin fragments under TEM and an increased amount of apoptosis. Adiponectin at a concentration of 20 μg/mL decreased the extent of HUVECs apoptosis. These results suggest that adiponectin may play a protective role in endothelial cells and alleviate their injury in sepsis. These findings are consistent with previous reports that adiponectin could exert vasculo-protective effects by inhibiting endothelial cell activation (28,29).

To gain insight into the protective mechanisms of adiponectin for endothelial cell injury, oxidative and ER stress of HUVECs were examined. The ER is the key organelle for cellular protein synthesis and processing, maintaining stability of the intracellular environment (30). Various extracellular (deprivation of calcium ions, hypoxia, and hyper-/hypo-osmosis) and intracellular (DNA damage or DNA replication stress) stresses can influence cell homeostasis (31). To adapt to such persistent unresolved stresses, misfolded proteins in the ER accumulate, and ER stress signals, such as IRE1α, are activated (32,33). Under physiological conditions, the luminal domains of IRE1α are bound to the ER molecular chaperone GRP78, which keeps it inactive. When unfolded (or misfolded), proteins accumulate in the lumen of the ER and IRE1α is activated through dissociation from GRP78. Upon activation, IRE1α induces signal transduction events that alleviate the accumulation of unfolded/misfolded proteins in the ER by increasing expression of GRP78 (34). Moreover, IRE1α activation can simultaneously activate the CHOP signaling pathway (35,36), resulting in cell apoptosis (37).

LPS, a constituent of the cell wall of gram-negative bacteria, is an activator of oxidative stress in sepsis, inducing the formation of ROS and MDA (11,12). ROS are associated with accelerated ER malfunction (13,14). The present results demonstrated that treating HUVECs with NAC down-regulated the phosphorylation of IRE1α, and the IRE1α inhibitor (STF-083010) reduced apoptosis of HUVECs as detected by TEM and flow cytometry.

Previous studies have shown that adiponectin alleviated ER stress in various cells and tissues, including human liver cells (38), mouse adipocytes (39), and rat smooth muscle cells (40). The current results showed that treatment with either adiponectin at a concentration of 20 μg/mL, or STF-083010, significantly decreased LPS-induced GRP78, CHOP, and cleaved caspase-12 expression, and apoptosis of HUVECs. This demonstrated the protective effects of adiponectin on endothelial cells.

In summary, we have confirmed for the first time that the administration of adiponectin may alleviate endothelial cell apoptosis by suppressing the ER stress IRE1α pathway. Therefore, endothelium protection with adiponectin may be a potential therapeutic strategy for coagulation dysfunction in sepsis. This will provide an early treatment reference strategy for clinical prevention of coagulopathy caused by sepsis. Although we demonstrated the protective effect of adiponectin on endothelial injury induced by sepsis, further mechanisms still need to be investigated, including finding new cell signaling pathways and new effective factors. In addition, clinical trials are needed to verify its effects on sepsis therapy.

## Supplementary Material

Click here to view [pdf].
